# Mechanistic Insights into Plant-Derived Exosomes, Their Cross-Kingdom Effects, and Potential Biomedical Applications in Skin Wounds Repair

**DOI:** 10.3390/plants15091286

**Published:** 2026-04-22

**Authors:** Adnan Amin, SeonJoo Park

**Affiliations:** Department of Life Sciences, Yeungnam University, Gyeongsan 38541, Republic of Korea; adnan.amin@yu.ac.kr

**Keywords:** tissue regeneration, TET8, IncRNA, multivesicular bodies, heat shock proteins

## Abstract

Plant-derived exosomes (PDEs) are gaining attention owing to their key implications in cross-kingdom communication, facilitating bioactive entities among plants and animals. PDEs are tiny nanoscale vesicles generally comprised of RNAs, proteins, and secondary metabolites and are involved in the regulation of physiological processes (immune modulation, cell regeneration, and stress response). An important feature of PDEs is to enable cross-kingdom regulation in skin wound repair. This is because PDEs can modulate several signaling pathways (PI3K-Akt, TGF-β, and mitogen-activated protein kinase) that further direct inflammatory, cell migratory, angiogenic, and extracellular matrix remodeling. Key features of PDEs, including modest immunogenicity, easy crossing of biological barriers, and natural biocompatibility, make them novel alternatives to synthetic wound-healing agents. Therefore, this review disparagingly examines the biogenesis, molecular composition, and diversified biological functions of PDEs, particularly with reference to potential implications in wound healing and overall skin health. The current challenges pertaining to PDE isolation, scalability, and bioavailability and regulatory hurdles for their clinical translation were also explored. In addition, the epigenetic effects of PDEs on human skin cells and wound healing are explained in detail. Finally, this review presents a comprehensive investigation of PDEs in skin wound repair, identifies research gaps, and outlines future directions for dermatological applications.

## 1. Introduction

The exosomes are tiny (30–150 nm in diameter), membrane-bound vesicles found in plants and animal cells and tend to expedite intercellular communication through the relocation of bioactive molecules [1]. These tiny vesicles are crucial for their major role in several physiological processes, such as cellular response to stress, tissue homeostasis, and immune modulation [2,3]. The biogenesis of such vesicles normally includes an initial inward budding/growth of the endosomal membrane that precedes the development of multivesicular bodies (MVBs) [4]. Several “cargo structures,” such as RNAs, proteins, lipids, and other molecules, are the main components of exosomes that can be transferred to recipient cells upon contact, modulating their behavior [5]. This “cargo transfer” feature of exosomes is considered very important in modern therapeutics because they can modulate cellular functions, for instance, signal transduction, gene expression regulation, and metabolism [6]. In this context, exosomes are increasingly recognized as natural delivery systems for regulatory nucleic acids, particularly microRNAs (miRNAs), small interfering RNAs (siRNAs), and other noncoding RNAs [7].

Plant-derived exosomes (PDEs), more precisely referred to as plant extracellular vesicles (EVs), have also been reported to enable communication within plants and animal cells (cross kingdoms) [8,9]. Similar to animal exosomes, these PDEs mainly contain siRNAs, proteins, lipids, and bioactive components (with slight variations) and play a key role in nutrition exchange, plant stress response, and diverse defense mechanisms [10]. As discussed earlier, PDEs act as vectors for cross-kingdom communication, influencing animal and human cells [11]. Accordingly, this review is organized to address five linked themes: PDEs’ structural characteristics, compositional variation across plant sources, roles in plant biology, cross-kingdom communication with animal cells, and therapeutic potential, particularly in skin wound healing and related dermatological applications. Furthermore, PDEs from *Ginseng*, *Arabidopsis thaliana*, *Curcuma longa*, etc., carry bioactive compounds, thereby affecting cellular functions in mammalian systems [12,13]. In regard to immune response, regulatory potential/metabolic pathways, and tissue regeneration capability of PDEs, interest is continuously building in their practical implications as therapeutic agents, particularly in dermatology [14].

Skin wound regeneration is an emerging area for developing PDE-based therapies [15]. Skin infection and wounds (particularly chronic wounds) are considered key clinical challenges, since existing therapeutic regimens are limited by resistance development, adverse effects, cost, and slow healing [16,17]. A more recent study revealed that PDEs greatly affect mitogen-activated protein kinase (MAPK), transforming growth factor-beta (TGF-β) and phosphoinositide 3-kinase–Akt (PI3K–Akt) pathways, thereby having major implications in the healing of chronic wounds and skin regeneration [18]. The natural capability of PDEs to enable targeted drug delivery to specific cells, along with their natural composition (proteins, lipids, and siRNAs), makes them a promising alternative to contemporary synthetic wound-healing agents [19].

In this review, PDEs were comprehensively analyzed, with a special focus on their cross-kingdom regulatory role and diverse molecular mechanisms through which they promote skin regeneration. In addition, commercial products, current challenges in PDE applications toward clinical settings, and parameters pertaining to stability, production, scalability, and safety are explained in detail. Through a detailed literature review, existing research gaps, predominantly in considering the molecular interactions between PDEs and human skin cells, were further identified. The aim was to examine in-depth the role of PDEs in skin health, particularly in the context of skin wound repair and cross-kingdom regulatory effects. This review summarizes the biogenesis, molecular cargo, and cross-kingdom functions of PDEs, with a specific focus on their role in skin wound repair and the challenges limiting their clinical application.

## 2. Literature Search Strategy

This narrative review was supported by a structured literature search. A well-structured literature search was accomplished using databases, which included Web of Science, PubMed, Scopus, and Google Scholar. All relevant manuscripts published in English (2020–2026) were included in the literature search. The “search terms” included “PDEs,” “plant EVs,” “miRNA,” “dermatology,” “exosome-like nanoparticles,” “cross-kingdom regulation,” “cargo proteins,” “angiogenesis,” and “exosomes.” The search priority was to include primary mechanism-based studies with in vitro or in vivo evidence. Regarding review articles, articles comprising interpretive comparisons and conceptual background were included. All literature was evaluated based on experimental outcomes, and the discussion was revised to differentiate animal evidence, in vitro, and translational data. A clear indication was marked in the review for contradictory or unclear findings.

## 3. Biological Properties of PDEs

PDEs are gaining recognition in modern skincare for their ability to carry bioactive compounds and their natural biocompatibility. Their low immunogenicity, along with their ability to cross biological barriers, makes them a highly attractive alternative to traditional therapies for skin regeneration [20].

### 3.1. Structural Features and Composition

PDEs share structural characteristics with their mammalian counterparts, although specific features may vary by plant source [21]. The mobility of “bioactive cargo” from PDEs is normally facilitated by their lipid bilayer membranes [22]. The key components of these membranes, i.e., glycolipids (glycosylceramides), sterols (sitosterol and stigmasterol), and phospholipids (phosphatidylcholine (PC), phosphatidylethanolamine (PE)), etc., mainly contribute toward fluidity and stability of the membrane [23,24]. In addition, several membrane proteins have been reported in PDEs, including TETRASPANIN 8 (TET8), TET9, PENETRATION 1 (PEN1), integrins, and RAB GTPases [1,25]. These diverse proteins mainly contribute to the biogenesis of exosomes, cellular adhesions, and trafficking across cells [26]. In addition, as “markers,” their presence as membrane proteins facilitates the isolation and characterization of PDEs [26].

**Figure 1:**
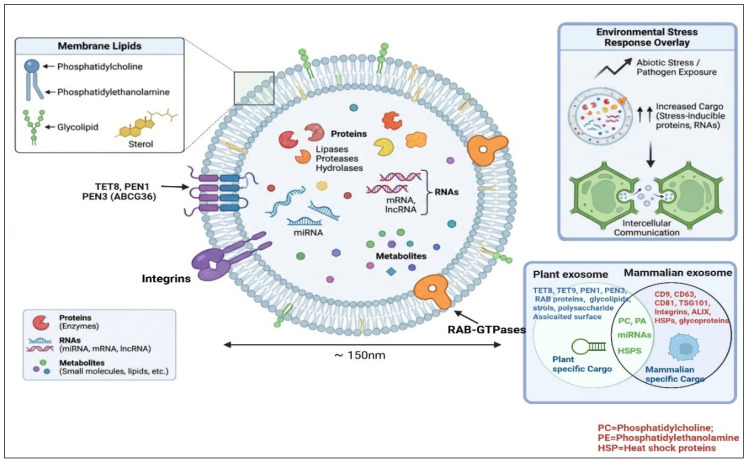
Detailed illustration of plant-derived exosomes, comparative analysis with animal exosomes, and environmental stress response overlay.

Earlier investigations revealed that the dynamic structure of PDEs generally changes in response to external biotics and biotic stress factors [27,28]. Such stressors induce modifications in RNAs and protein composition, which allows cellular communication with neighboring or even cells of other organisms [29,30]. Thus, the regulation of molecular signals and stability during secretion mainly relies on the overall structural integrity of PDEs (Figure 1).

### 3.2. Composition and Biological Functions

Key biological functions of PDEs mainly depend on their components, namely, RNAs, cargo proteins, secondary metabolites, and lipids [31]. Among RNAs, diverse types, including long noncoding RNAs, siRNAs, and microRNAs, have been reported with varied functions [32,33]. Among these, microRNAs are very important for gene silencing, thereby binding to complementary mRNA sequences in recipient cells [10,29]. In addition, cross-kingdom regulation is attributed to the presence of siRNAs in PDE that influence gene expression in mammalian cells upon delivery [34].

Various cargo proteins, such as vacuolar-type ATPase subunits, TET8, PEN1, PENETRATION 3 (PEN3), PM1-INTERACTING PROTEIN 4 (RIN4), ALIX homologs, aquaporins, syntaxins, fasciclin-like arabinogalactan proteins, germin-like proteins, calreticulins, and components linked to vesicle secretion, such as EXO70 family proteins and VAP27, have been reported in PDEs [35,36,37]. Several enzymes, such as lipases, proteases, and hydrolases, have also been reported in these cargo proteins [38]. Cargo proteins in PDEs, in addition to their implications in biogenesis, play a key part in trafficking, signal transduction, and various cellular interactions [24,39]. As discussed in an earlier section, typical structural features of PDEs are mainly reliant on different types of lipids, that is, intricate formation, fusion, and stability of exosomal interactions with recipient-cell membranes [40].

**Figure 2:**
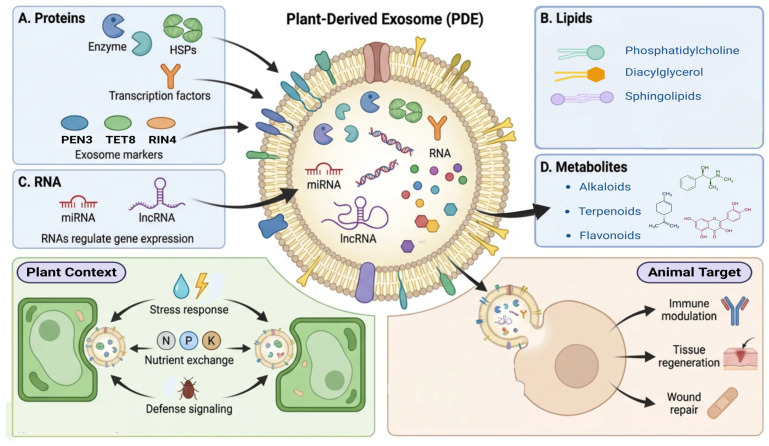
Composition and biological functions of PDEs.

Ultimately, the most important components of PDEs are secondary metabolites [41]. Diverse secondary metabolites, such as alkaloids, terpenoids, saponins, and flavonoids, have been reported, which are specific to the plant/species [12,42]. The importance of PDEs against several health concerns revolves around the transport of these secondary metabolites to animal cells, where they may exert therapeutic effects (antioxidant, anti-inflammatory, and anticancer) [43,44]. The exosome-mediated transfer of secondary metabolites is of particular interest because it provides a clear understanding of how PDEs influence various cellular pathways in human cells (Figure 2).

The biological functions of PDEs vary depending on plant species and secondary metabolites, with roles in stress response, nutrient exchange, and defense signaling [45]. One of the key functions of PDEs is cell-to-cell communication, which is generally responsible for regulating the response of plants toward biotic and abiotic stressors (pathogens, drought, salinity, etc.) [46,47]. This communication helps plant cells to give a combined and coordinated response to external stress. Moreover, PDEs can allow and facilitate the exchange/transport of nutrients among diverse plant cells, thereby supporting overall cellular homeostasis [9,48]. PDEs also play an active and key role in cross-kingdom regulation, enabling immune modulation, cell migration, and tissue regeneration in animal cells [9]. The capability of PDEs to deliver bioactive moieties, microRNAs, and proteins to recipient cells is crucial for their therapeutic potential, particularly in skin health and wound regeneration (Figure 2, Table 1) [49].

PDEs may contribute to nutrient-related communication by carrying nutrient transporters, proton ATPases, aquaporins, metabolites, and small RNAs linked to rhizosphere nutrient regulation, although current evidence supports regulatory rather than bulk nutrient transport functions [50].

**Table 1:** Major molecular components of plant-derived exosomes and their reported biological relevance.

Molecular Category	Representative Molecules	Proposed Biological Relevance	Ref
small RNAs	miRNAs and siRNAs	post-transcriptional gene regulation and potential cross-kingdom signaling	[51,52]
long noncoding RNAs	lncRNAs	broader regulation of gene expression and stress-associated signaling	[53,54]
structural/membrane-associated proteins	TET8, PEN1, PEN3, aquaporins, syntaxins, and fasciclin-like arabinogalactan proteins	vesicle identity, membrane organization, cargo packaging, and intercellular interaction	[24,55]
functional/trafficking-related proteins	ALIX homologs, RIN4, EXO70 family proteins, VAP27, and vacuolar-type ATPase subunits	vesicle biogenesis, cargo sorting, trafficking, secretion, and signaling	[24,56]
enzymatic proteins	lipases, proteases, and hydrolases	catalytic remodeling and possible modulation of recipient-cell physiology	[56]
lipids	glycosylceramides, phosphatidylcholine, phosphatidylethanolamine, sitosterol, and stigmasterol	membrane stability, vesicle formation, fusion, and cargo protection	[24,40]
secondary metabolites	flavonoids, terpenoids, alkaloids, saponins, and phenolic compounds	antioxidant, anti-inflammatory, antimicrobial, and regenerative activities	[43,57]

### 3.3. Synthesis Pathways of PDEs

Similar to other eukaryotic cells, the initiation of endocytic pathways is the main mechanism involved in PDE synthesis [58]. In this pathway, an “early exosome” arises from the initial invagination of the plasma membrane [59]. These “early exosomes” were then further modified to “late exosomes” after maturation, resulting in MVB formation [38,60]. These MVBs are further categorized by the development of intraluminal vesicles (ILVs) in the late endosomal compartment [61]. Thus, PDE formation is mainly dependent on the plasma membrane’s inward budding since MVBs then fuse with the cellular membranes and enable ILV release into the extracellular space as exosomes [62].

MVB trafficking and the endosomal sorting complex required for transport (ESCRT)-related endosomal system are the main controlling factors of PDE biosynthesis [63]. Here, during ILV formation, cargo sorting and remodeling of the membrane are accomplished using conserved ESCRT components. The MVB dynamics are supported by ALIX-related (homologs) and VPS4/SKD1-associated functions [37,64]. Nevertheless, the detailed and exact molecular framework of PDE biogenesis remains less well defined than that of mammalian exosomes, and the relevance of canonical markers (e.g., TSG101) requires further validation in plant systems [65,66].

**Figure 3:**
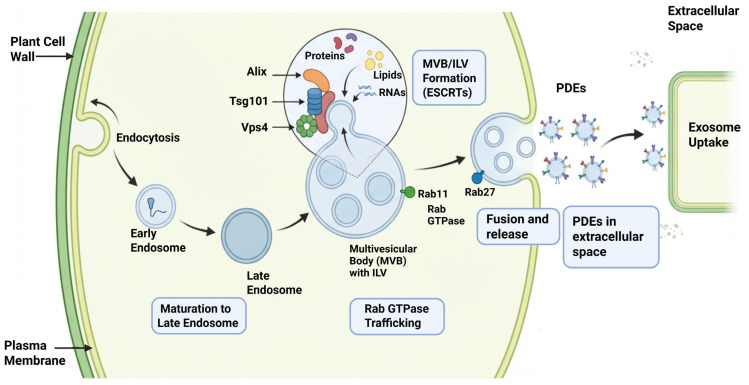
An illustrated overview of Biosynthetic pathway in PDEs, endophytic trafficking, ESCRT mechanism, exosome release and uptake mechanism. The depicted pathways and hormone structures are illustrative and representative, and do not represent a complete regulatory network.

PDE production is generally linked to Rab GTPase–regulated membrane trafficking pathways rather than to a clearly established Rab27-dependent mechanism [67]. Plant Rab-A GTPases, which are functionally related to the Rab11 group in other eukaryotes, regulate trafficking between the trans-Golgi network, endosomal compartments, and plasma membrane and are therefore likely to contribute to EV secretion [68,69]. In addition, plant MVBs can interact with the cellular membranes and thereby release ILVs into the apoplast, providing a plausible route for the extracellular release of exosome-like vesicles. However, the specific molecular regulators of this final fusion step remain less completely defined in plants than in mammalian systems (Figure 3) [70].

At present, most mechanistic studies have focused on angiosperms, particularly *A. thaliana*, tomato, and several edible or medicinal species. Conversely, direct evidence from gymnosperms remains scarce, and robust side-by-side comparisons between gymnosperms and angiosperms are largely unavailable. Therefore, although phylogenetic differences in vesicle composition, marker conservation, or secretion dynamics are biologically plausible, current data do not yet justify strong conclusions regarding lineage-specific EV biogenesis [65,71]. This represents an important knowledge gap in the field and warrants targeted comparative studies.

A distinct case is the root system, where EVs are secreted into the rhizosphere and may mediate interactions with surrounding microorganisms. Root-derived vesicles have been isolated from tomato root exudates, contain defense-related proteins, and inhibit fungal spore germination and germ tube development, supporting a role in extracellular antimicrobial defense [72]. Recent work further indicates that roots can release EV-associated small RNAs into the rhizosphere. In this case, these may suppress pathogens and favor beneficial microbial taxa, thereby contributing to disease-suppressive soil functions and microbiome assembly [73]. These findings are consistent with the broader view that rhizosphere EVs may influence nutrient cycling, microbial recruitment, and communication at the plant–soil interface [50].

### 3.4. Sources and Extraction Methods

PDEs have been isolated from a broad range of species rather than from “edible and medicinal plants” as a generic category. PDEs are highly abundant in leaves, roots, fruits, and juices of edible and medicinal plants [12,74]. Plant tissues with intense metabolic activity, such as leaf mesophyll and root tissues, tend to produce more PDEs owing to their roles in environmental responses and intercellular communication [75]. Across these sources, the juice or sap of fruits and roots and apoplastic fluid or extracts in the leaves often provide a fluid matrix rich in exosome-like vesicles. For instance, *A. thaliana* leaf apoplastic fluid is extensively used as a model system for isolating clean, intact PDEs [63], and the protocol for extracting these exosomes from *Arabidopsis* leaves is well documented, including steps such as apoplastic washing fluid collection and density gradient fractionation [76]. Similarly, *Panax ginseng*, a high-metabolism medicinal plant, yields substantial exosome-like nanoparticles from its root, stem, and leaf tissues, with optimized isolation methods improving purity and colloidal stability [77].

**Figure 4:**
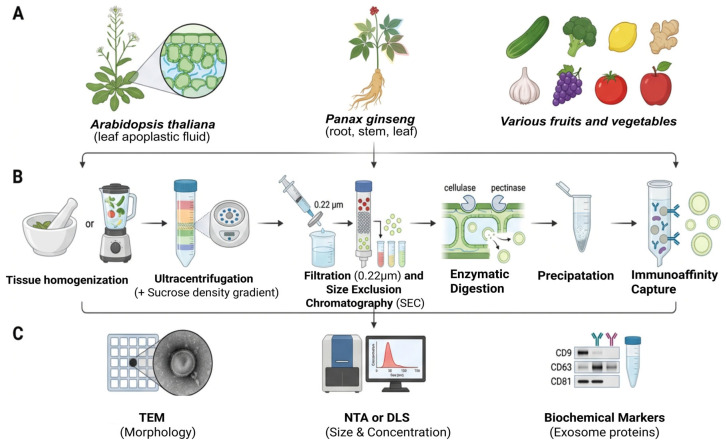
A summary of common sources including various plants and vagetables (**A**), common extraction schemes including tissue homogenization, ultracentrifugation, Filteration and size exclusion chromatography, Enzymatic digestion, followed by precipitation and immunoaffinity capture (**B**), and advanced tools like transmission electron microscopy (TEM), Nanoparticle Tracking Analysis (NTA) and Dynamic Light Scattering (DLS) (**C**) for the characterization of PDEs.

Other edible plant sources identified in the literature as yielding notable levels of PDEs include cucumber, broccoli, lemon, ginger, garlic, grape, and tomato, in recent isolation strategy studies [78]. Fruits such as apples (*Malus domestica*) also serve as scalable sources of EVs; apple-derived EVs have been isolated and characterized for their protein, lipid, and nucleic acid cargoes, highlighting the broad distribution of PDEs across different plant tissues and organs (Figure 4) [79].

PDE extraction typically begins with the homogenization of selected plant tissues in appropriate buffers, followed by sequential purification steps to isolate exosome-enriched fractions [80]. Ultracentrifugation remains the most widely utilized method: differential centrifugation removes cellular debris and pelleted vesicles, and high-speed ultracentrifugation (often using a sucrose density gradient) captures the nano-sized exosomal fraction [77]. This method exploits differences in particle size and density to enrich EVs. Filtration through a 0.22-µm membrane or smaller is often used before ultracentrifugation to remove larger particles and streamline vesicle isolation [14]. Density gradient centrifugation further refines exosome purity by separating vesicles based on buoyant density. Moreover, size-exclusion chromatography (SEC) can be applied to fractionate PDEs by size with minimal shear stress, preserving morphological integrity [76,81].

In addition to physical separation techniques, enzymatic cell wall digestion has been developed to increase yield by releasing EVs embedded within the rigid plant cell wall matrix [82]. Plant cell walls can be digested with enzymes targeting cellulose and pectin, thereby liberating EVs into solutions for subsequent collection, improving isolation efficiency, especially for tissues with robust cell walls, such as roots [80,83]. Precipitation-based methods (e.g., polyethene glycol or commercial kits) offer simpler procedures but generally yield lower specificity than ultracentrifugation and SEC [84]. Finally, immunoaffinity capture using antibodies targeting conserved exosomal markers (such as plant tetraspanins) enables selective enrichment of exosome subpopulations [80]; however, this approach requires knowledge of species surface proteins and reliable antibodies.

PDEs are generally characterized using several advanced techniques to determine size distribution, concentration determination, and understanding of various biochemical markers. Some of the commonly used techniques include transmission electron microscopy, dynamic light scattering, and nanoparticle tracking analysis (Figure 4) [85,86,87].

The source material influences both vesicle yield and composition. For example, the leaf apoplastic fluid is useful for studying relatively defined EV populations in intact plant tissues, whereas fruit homogenates or juices often provide higher material yield but may contain more heterogeneous vesicle-like particles. Similarly, root-derived vesicles and root exudate-associated vesicles are particularly relevant to rhizosphere signaling and plant–microbe interactions [72]. A comparative summary of representative species and plant parts reported as vesicle sources is provided in Table 2.

## 4. Cross-Kingdom Regulation of PDEs

### 4.1. Small RNAs and Gene Regulation

In the context of cross-kingdom regulation in PDEs, the role of small miRNAs is of primary concern [96]. These noncoding RNAs bind with complementary mRNA sequences and thereby regulate gene expression, translation repression, and degradation of mRNAs [97]. Plant exosomes, rich in miRNAs, can affect the gene expression of mammalian cells [10,98]. For instance, the *Centenella asiaitica* miRNA (isolated from exosomes) can regulate the expression of Peak1 pertaining to gut microbiota regulation, metabolic hemostasis, and systemic immunity [99]. Several examples from plants clearly explain the role of miRNAs in gene expression; for instance, in *P. ginseng* (ginseng), miR-155-156 expression regulates macrophage activation, immune modulation, and tissue regeneration [100]. These key features of fmiR-155-156 are considered very important in cross-kingdom regulation, particularly in the context of tissue regeneration.

### 4.2. Proteins and Immune Modulation

The proteins in PDEs are another important entity that can generally facilitate cross-kingdom regulation [101], although their mechanistic understanding compared with mammalian exosomes remains unclear [102]. Reported protein cargoes include membrane-associated proteins, such as TET8, syntaxins, aquaporins, calreticulins, germin-like proteins, and vacuolar-type ATPase subunits, which are linked to vesicle identity, membrane organization, and extracellular targeting [71]. For instance, in *A. thaliana*, adequate evidence reveals that key heat shock proteins, including HSP70 or HSP90, can directly modulate mammalian cytokine signaling through pattern recognition receptors (PRRs) [36,71,88]; however, a direct experimental validation is still needed. Another validated report is related to rice, in which defense proteins (OsDUF26, OsJLL1, OsESP1, and OsFW2.2) were transferred to a fungus (*Rhizoctonia solani*) that contributed toward disease suppression. In general practice, evidence for immunomodulation is sturdier for whole PDE-based preparations than for individual protein cargo. PDEs from *Opuntia ficus indica* significantly reduced interleukin (IL)-8, IL-6, and tumor necrosis factor (TNF)-α levels in stimulated human cells and promoted wound repair [103]. Similarly, EVs obtained from tomato fruit presented anti-inflammatory activity with additional enhancement in activity upon loading with curcumin [104]. Thus, a clear comparable primary evidence for specific PDE proteins acting in mammalian recipient cells remains scarce.

PDEs also contain trafficking- and secretion-related proteins, such as ALIX-associated proteins, EXO70 family proteins, and VAP27-related factors, which are relevant to vesicle biogenesis, cargo sorting, and secretion. In addition, defense-associated proteins, such as PEN1, PEN3-related proteins, and RIN4-associated components, have been linked to plant immune responses and may contribute to extracellular defense signaling [71,105].

### 4.3. Lipids and Cell Membrane Regulation

Nevertheless, the role of various lipids obtained from PDEs is also critical. Several structurally imperative lipids, such as sphingolipids, sterols, phospholipids, and sphingolipids significantly contribute toward cargo delivery in addition to vesicle stability and membrane integrity [88]. Several examples support evidence of the regulatory role of lipids in cell membrane stabilization. For instance, the grapefruit (*Citrus paradisi*)-based nanovesicles reduced colitis in mice by lowering IL-1β and TNF-α expression [106]. Similarly, orange juice-based nanovesicles modulate gut barrier-associated functions [107]. More recently, EVs rich in phosphatidic acid were proven to improve epithelial transcytosis through MAPK/ERK1/2 signaling [108]. Further, in *A. thaliana*, although EVs contribute to plant defense and show substantial heterogeneity, direct evidence that their lipid cargo specifically modulates mammalian macrophage or dendritic cell function remains lacking [36].

### 4.4. PDEs and Skin Health

Plant exosomes are crucial in promoting skin regeneration, mainly through TGF-β signaling, which regulates collagen production and fibroblast differentiation [109]. Among these, TGF-β/SMAD signaling is particularly important because it promotes fibroblast activation, collagen synthesis, and extracellular matrix deposition, which are required for wound closure and tissue remodeling. In this context, EVs from *P. ginseng* were reported to enhance skin regeneration through the activation of TGF-β/SMAD signaling, supporting a role in dermal repair and matrix restoration [110]. Thus, this finding reflects applications in skincare and dermatology. Similarly, *Aloe vera*-derived exosomes modulate TGF-β signaling and promote angiogenesis, which is vital for supplying oxygen and nutrients to regenerating tissues [111]. Similarly, exosomes derived from Kale (Brassicaceae) were found to inhibit TGF-β signaling and promote antiaging effects [112]. Additionally, plant exosomes influenced keratinocyte proliferation and migration, which are key to the re epithelialization phase of wound healing. Typical examples include wheat- and grapefruit-derived exosomes [113].

The MAPK pathway is another major node through which PDEs may affect skin physiology [114]. MAPK signaling regulates keratinocyte proliferation, migration, stress responses, and inflammatory signaling and is therefore highly relevant to reepithelialization [109]. Current evidence suggests that PDE cargo, such as lipids and bioactive phytochemicals, can influence MAPK-associated responses, thereby supporting epithelial repair, cellular adaptation to injury, and wound closure [115].

In addition to wound healing, PDEs may have broader relevance in dermatology because their cargo can influence inflammation, oxidative stress, epithelial repair, and extracellular matrix regulation [116]. Accordingly, emerging studies have suggested potential applications in photoaging, inflammatory skin disorders, and barrier-associated skin damage; however, the mechanistic and clinical evidence for wound repair remains insufficient [117,118].

PDEs have been investigated for skin-related applications mainly in in vitro and preclinical in vivo studies. In vitro studies have shown that these vesicles can promote keratinocyte and fibroblast migration, reduce oxidative stress, and modulate inflammatory signaling, which are relevant to wound repair and skin regeneration [119]. In animal models, particularly murine wound-healing studies, plant-derived vesicles accelerate wound closure, enhance collagen deposition, and support angiogenesis, thereby providing stronger evidence for therapeutic potential beyond cell-based observations [120,121]. By contrast, clinical evidence in humans remains limited, and the current literature does not yet support firm conclusions regarding clinical efficacy [122].

Regarding administration, PDEs in wound-healing studies have been evaluated predominantly by local or topical application at the wound site. Therefore, the current evidence supports a promising preclinical role for PDEs in skin repair; however, further controlled clinical studies are needed to confirm their therapeutic value [121,123].

### 4.5. Epigenetic Influence of PDEs

PDEs may influence epigenetic processes in human cells through DNA methylation, histone modification, and regulation of noncoding RNAs (miRNAs), thereby modulating gene expression at a deeper level [124,125]. The miRNAs carried by PDEs may target genes involved in cancer therapy, influencing how human cancer cells respond to this exposure. For example, *B*. *javanica*–derived exosomes greatly influenced the underlying molecular mechanisms of triple-negative breast cancer caused by miRNAs’ modulatory function (Table 3) [126].

## 5. Therapeutic Applications of PDEs in Skin Health

PDEs have intrinsic biocompatibility and low immunogenicity and are rich in biologically active cargo (lipids, proteins, small RNAs, and phytochemicals) that can modulate multiple cellular processes necessary for dermal regeneration and homeostasis [139].

### 5.1. Antiaging Effects

PDEs are well known for their antiaging effects, which are mainly attributed to antioxidative, dermal cell proliferation, and extracellular matrix production [140]. For example, PDE preparations from grapes [141] and citrus fruits [142] have been characterized for their antioxidant capacities and suggested for their potential to mitigate oxidative stress, a major contributor to skin aging [143]. Similarly, nanovesicles from the Goji berry plant were reported to affect aging-related muscle loss by activating the AMPK/sirtuin 1 (SIRT1)/peroxisome proliferator-activated receptor-γ coactivator 1-α pathway [126]. Although specific clinical trials on PDEs for aesthetic skin rejuvenation remain limited, certain reviews highlight their capacity to neutralize reactive oxygen species (ROS) and modulate fibroblast activity, offering mechanistic plausibility for antiaging effects [144]. The antioxidative features of PDEs imply a protective role in keratinocytes and fibroblasts, thereby avoiding oxidative DNA damage. This effect is mainly due to the presence of secondary metabolites and has useful implications in age-associated dermal degeneration [12].

### 5.2. Wound Healing and Tissue Regeneration

Multiple preclinical studies have demonstrated that PDEs exhibit therapeutic effects on wound healing by promoting cell proliferation, migration, extracellular matrix deposition, and angiogenesis [145]. Herein, a commercial PDE product, Elysee Exosome PDRN Ampoule (DermaFirm, Republic of Korea), comprising *Centella asiatica* leaf vesicles, demonstrated wound closure and tissue regeneration effects on mice [146]. Similarly, orange-derived EVs (*Citrus sinensis*, variety Tarocco) are reported to effectively accelerate in vivo wound closure in healthy and diabetic mice [147]. Furthermore, exosomes increased cell viability and cell migration while reducing intracellular ROS levels in a dose-dependent manner in HaCaT cells [148]. Nevertheless, exosomes derived from wheat, mango, and ginger have been reported to increase keratinocyte viability and migration in vitro and enhance endothelial cell tube formation, indicating improved healing potential [149,150].

### 5.3. Anti-Inflammatory and Antioxidant Effects

One of the most extensively described bioactivities of PDEs is their antioxidant and anti-inflammatory capacity [151]. This outstanding activity is mainly attributed to the presence of flavonoids, phenolic acids, and other phytochemicals in PDEs that can effectively reduce oxidative stress in recipient cells [152]. Reviews note that PDEs deliver phytochemical antioxidants that, in vitro, counteract excessive ROS, a key driver of both cellular aging and chronic inflammation in mammalian tissues [144]. Several examples of PDEs with significant antioxidant capacity have been reported. For instance, *Catharanthus roseus* (L.)–based exosomes strongly stimulated the secretion of TNF-α, activated NF-κB signaling pathway, and increased the expression of the hematopoietic function-related transcription factor PU.1, in vitro and in vivo [153]. Similarly, PDEs from *Carica papaya* L. fruit decreased nitric oxide production and facilitated the downregulation of mRNA expression of proinflammatory cytokine genes (IL-1β and IL-6) and an upregulation in mRNA expression of anti-inflammatory cytokine genes (IL-10). This was attributed to the presence of 2,3-dihydro-3,5-dihydroxy-6-methyl-4H-pyran-4-one in PDEs [154]. Similarly, PDEs derived from edible plants (*Citrus limon*) are reported to suppress proinflammatory mediators in model systems by interacting with innate immune signaling processes [155]. Several other fruits, vegetables, and herbs were reported to possess significant anti-inflammatory potential [12].

### 5.4. Treatment of Skin Disorders

PDEs are considered important in the management of skin-related disorders owing to their oxidative stress–lowering and modulatory effects on immune signaling. Several investigations have confirmed the beneficial effects of PDE on skin; for instance, tomato (*Solanum lycopersicum*)-derived nanovesicles boost keratinocyte and fibroblast migration in vitro, thereby accelerating wound closure [119]. Similarly, EVs from *Opuntia ficus indica* significantly repressed IL-6, IL-8, and TNF-α and stimulated fibroblast migration, demonstrating the regulation of inflammatory signaling and skin tissue repair [156](V In another case, EVs from *Perilla frutescens* inhibited RSL-3-induced ferroptosis, resulting in decreased expression of antioxidant and lipid peroxidation-related factors [157]. Nevertheless, the EVs derived from shallot and garlic presented promising outcomes in psoriasis. This effect was mediated by acting on IL-17 signaling modulated by the erythroid-2-related factor 2 (NRF2) pathway [158]. Nevertheless, exosomes loaded with secondary metabolites have also been effective against psoriasis. As noted, the epigallocatechin-3-gallate nanoparticle–loaded exosome showed significant effects on psoriasis-related markers (IL-6, IL-4, Bcl-2, Bax, NF-κB, and CDC25B) [159].

### 5.5. Photoaging

PDEs have effectively been reported against skin photoaging. In an investigation, lavender-derived exosome-like nanoparticles effectively promoted collagen synthesis and alleviated photoaging in ICR mice [160]. Similarly, *Aloe vera*-derived exosome-like nanoparticles demonstrated significant antioxidant and anti-inflammatory properties by scavenging ROS, activating the Nrf2/ARE pathway, and enhancing collagen synthesis, thereby protecting against photoaging [161]. Similarly, in vivo experiments with ICR mice revealed that *Olea europaea* leaf–derived exosome-like nanovesicles prevented skin damage caused by UV radiation, including collagen and elastin degradation [162].

### 5.6. Clinical Studies and Trials

Several clinical studies and trials on PDEs are in the early stages and are investigating the effects of PDEs on skin care, specifically wound healing and antiaging. In a clinical trial (randomized controlled trial), they investigated the therapeutic potential of an *Echinacea purpurea* and *Cucumis sativus* exosome–derived formulation for treating male pattern alopecia. These results highlight the efficacy of exosome-based therapy in promoting follicular regeneration during androgenic alopecia [163]. Similarly, a phase I clinical trial (NCT01668849) investigated grape-derived EVs for the prevention of oral mucositis. This trial validated PDEs being pursued in human settings for mucosal/epithelial regeneration [164]. Although the clinical data pertaining to human clinical trials in PDE development are limited, several commercial products available in the market are considered in various dermatological cases (Table 4).

### 5.7. In Vitro Investigations

*In vitro* studies have shown that PDEs enhance keratinocyte and fibroblast migration, increase cell viability, and reduce oxidative stress, supporting roles in re-epithelialization and tissue repair [103,119]. Preclinical in vivo studies, mainly in murine wound models, have further indicated accelerated wound closure, improved collagen deposition, and enhanced angiogenesis following local administration of plant-derived vesicles [120,121]. By contrast, clinical evidence remains limited; therefore, current translational conclusions are preliminary [165].

## 6. Challenges and Future Directions

Notwithstanding the auspicious therapeutic potential of PDEs, numerous challenges need careful consideration before widespread clinical application.

One of the key challenges is “isolation” and “purification” of PDEs, which mainly relates to the lack of any universal standardization protocols [166]. For instance, ultracentrifugation is the most widely used method; however, it remains challenging owing to low output and specialized equipment [167]. Conversely, concerns about low purity persist in ultrafiltration, although it is rapid and cost-effective [168].

Moreover, variation in different batches of isolated PDEs needs attention. This major factor mainly depends on diversification in plant species, variable growth conditions, and different extraction schemes. These factors contribute toward diverse chemical composition and overall quality of isolated exosomes [169]. The bioavailability and stable formulation design in PDEs are of great concern, directly related to overall effects and clinical applications. Here, the interaction of key exosome components (miRNAs, proteins, and lipids) with excipients of formulation is another aspect of concern [170].

Nevertheless, ethical and regulatory challenges must be addressed carefully. Despite their natural origin and less immunogenicity, a detailed safety analysis during clinical trials limits their commercialization [171]. Indeed, to ensure public safety, the U.S. Food and Drug Administration and European Medicines Agency place stern regulatory guidelines for nanomedicines and biological products that PDEs must adhere to. To the best of our knowledge, regulatory guidelines related to PDEs are restricted, making the final approval of such products limited. Also, long-term clinical trials on their safety and efficacy in humans are scarce.

## 7. Integration with Emerging Technologies

The therapeutic development of PDEs may be substantially strengthened by integration with emerging technologies that improve mechanistic resolution, formulation design, and delivery efficiency. Gene-editing approaches, such as CRISPR-Cas systems, may help identify and validate genes involved in vesicle biogenesis, cargo loading, and stress-responsive secretion, thereby improving the mechanistic understanding of how PDEs are formed and how their bioactive content can be modulated in source plants. Such tools may also support engineering strategies aimed at enriching selected RNAs, proteins, or metabolites within vesicle populations [172]. Artificial intelligence and machine learning may further accelerate PDE research by enabling the analysis of complex omics datasets, the prediction of bioactive cargo candidates, and the selection of plant sources with favorable vesicle composition. These computational approaches may also be useful for optimizing formulation variables, stability conditions, and source-to-function relationships that are otherwise difficult to resolve experimentally across diverse plant systems [173]. Such large language models can help in predicting optimized and effective combinations of PDEs with other therapeutic agents synergistically. Another promising area for integration is nanotechnology, which can improve PDE-based delivery systems. Such integration can be further considered useful in protecting PDEs from degradation [174].

Nanotechnology represents another important area for integration to enhance PDE stability, topical delivery, and controlled release. Hybrid systems that combine PDEs with biomaterials, hydrogels, microneedles, or nanoformulations may enhance skin penetration [15], protect vesicle cargo from degradation, and increase retention at wound sites or inflamed skin regions. Collectively, these emerging technologies may support the transition of PDEs from descriptive biological entities to more standardized and clinically adaptable therapeutic platforms.

## 8. Conclusions

PDEs present auspicious frontiers in the management of skin-related health concerns. PDEs are considered promising therapeutic candidates because of their modulatory effects on inflammation, promotion of cellular migration, and enhanced angiogenesis. This makes PDEs promising in the management of skin conditions, particularly chronic wounds that are difficult to treat with conventional therapies. However, as this review highlighted, significant challenges remain in optimizing their isolation, stability, and bioavailability. Addressing these issues, along with regulatory and ethical considerations, will be essential for the clinical translation of PDEs. With continued research, PDEs could become a cornerstone of regenerative medicine, offering safe and effective treatments for a wide range of skin injuries and disorders. Future research should also focus on the mechanistic understanding of how PDEs influence human skin cells at the genetic and epigenetic levels. Although the current understanding of their effects on inflammation and cell migration is expanding, further investigations are needed to understand the mechanisms by which proteins, miRNAs, and lipids within PDEs regulate gene expression. Such research could reveal novel therapeutic targets for PDEs, enhancing their effectiveness in wound healing. Additionally, clinical trials are necessary to assess the safety and efficacy of PDE-based therapies, particularly in chronic wound models and human patients. Current limitations include the lack of methodological standardization, variability in plant sources and vesicle composition, incomplete mechanistic validation, and the scarcity of robust clinical evidence. Future studies should focus on standardized production and characterization protocols, mechanistic studies linking specific cargo to biological outcomes, improved formulation and delivery strategies, and controlled clinical trials to support translation into therapeutic practice.

## Figures and Tables

**Table 2:** Representative plant species reported as sources of extracellular vesicles.

Plant Species	Common Name	Plant Part Used for Vesicle Isolation	Key Components/Effects	Notes/Relevance	Ref
*Arabidopsis thaliana*	arabidopsis	leaf apoplastic washing fluid	small RNAs, defense-related proteins, and lipids	model EV system	[71,88]
*Solanum lycopersicum*	tomato	fruit tissue and root exudates	proteins, lipids, and metabolites; anti-inflammatory	fruit and root EV sources	[72,89]
*Panax ginseng*	ginseng	root, stem, leaf, and callus-derived materials	ginsenosides, proteins, lipids, and miRNAs/antioxidant and anti-inflammatory	medicinal EV source	[90,91]
*Zingiber officinale*	ginger	rhizome/rhizome juice	gingerols, shogaols, lipids, and small RNAs/anti-inflammatory and antioxidant	rhizome-derived EV source	[89,91]
*Citrus paradisi*	grapefruit	Fruit juice	lipids, flavonoids, and membrane-associated bioactives/anti-inflammatory and antioxidant	widely used fruit EV source	[92]
*Citrus limon*	lemon	fruit juice	flavonoids, lipids, and small RNAs/anti-inflammatory and antioxidant	citrus EV source	[93]
*Vitis vinifera*	grape	fruit juice/berry material/cell culture	polyphenols, stilbenoids, lipids, and miRNAs/antioxidants	bioactive fruit EV source	[94]
*Malus domestica*	apple	fruit tissue/juice	polyphenols, lipids, and vesicular metabolites/antioxidants	scalable fruit EV source	[93]
*Aloe* spp./*Aloe vera*	aloe	leaf or peel-derived material	lipids, proteins, and polysaccharide-related metabolites/anti-inflammatory and antioxidant	skin-relevant medicinal EV source	[90,93]
*Coffea arabica*	coffee	cell suspension culture medium	proteins and EV-associated cargo identified by proteomic characterization	in vitro culture EV source	[95]

**Table 3:** Cross-kingdom regulation via plant-derived exosomes: implications for skin regeneration and immune modulation.

Plant Species/Source	Exosome Cargo	Biological Impact	Key Mechanism	Ref
*Arabidopsis thaliana*	Small RNAs, proteins, and lipids	Plant immunity	Stress-responsive EV signaling	[36,127]
*Panax ginseng*	Lipids, proteins, and metabolites	Wound healing and skin repair	ERK/AKT, TGF-β/SMAD	[128,129,130]
*Curcuma longa*	Curcumin, lipids, and proteins	Anti-inflammatory activity	NF-κB inhibition	[131,132]
*Vitis vinifera*	miRNAs, proteins, and stilbenoids	Skin protection and regeneration	Epithelial repair signaling	[133,134]
*Aloe saponaria*	Lipids, proteins, and metabolites	Chronic wound healing	Anti-inflammatory and angiogenic	[135]
*Dendrobium officinale*	Lipids, proteins, and RNAs	Wound healing and angiogenesis	Akt/eNOS activation	[136,137]
*Solanum lycopersicum* (tomato)	Nanovesicles and bioactive cargo	Wound closure	Cell migration promotion	[119]
General PDEs	RNAs, proteins, and lipids	Immune modulation and tissue repair	Cargo-mediated signaling	[93,138]

**Table 4:** Commercially available PDE-based cosmetic products.

Product Name	Exosome Type	Plant Source	Country	Key Functions/Effects
EXO	E™ Revitalizing Complex	Plant exosome-like vesicles	Austria/UK	skin rejuvenation, hydration, and regeneration
EXOME (식물줄기세포 유래 엑소좀)	plant stem cell–derived exosomes	*Centella asiatica* and other plants	UK	anti-aging and skin vitality
Inkey List Exosome Hydro-Glow Complex Serum	exosome-like vesicles	*Centella asiatica*	Switzerland	collagen synthesis, antiaging, and hydration
ExoBloom by Morganna’s Alchemy	plant stem cell–derived exosomes (Goji berry)	*Lycium barbarum* (Goji)	UK	skin regeneration and antiaging
Plant-Based Aloe Exosome Active	exosome-like vesicles	*Aloe vera*	Malaysia	hydration, barrier support, and anti-inflammatory properties
PhytoCellTec™ Exosomes (Mibelle Biochemistry)	plant stem cell–derived vesicles	*Lycium barbarum* (Goji)	Switzerland	antiaging, boosts collagen and elastin levels
Naolys ExoCell^®^ Plant Exosome Biotech Platform	custom plant exosome platform	Various botanical species	France	skin barrier support and regeneration
EXO DRF AZ (Plant exosome for cosmetics)	plant exosome-like vesicles	Multiple herbs and fruits	Not specified	hydration and skin barrier repair
Creative Biostructure Plant Exosome Ingredients	plant exosome active ingredient	Various botanical sources		anti-aging, hydration, and antioxidant
Dragon Fruit and Tulsi Exosome Actives	plant exosome ingredients	*Hylocereus undatus* (dragon fruit) and *Ocimum tenuiflorum* (Tulsi)	Various	antioxidant, anti-inflammatory, and regeneration
Grand Ingredients Apple Exosome Active	exosome-like vesicles	*Malus domestica* (apple)	Various	anti-aging, collagen support, and skin elasticity
VYTRUS Plant Stem Cell Exosomes (*Centella* and *Curcuma*)	plant stem cell–derived exosomes	*Centella asiatica* and *Curcuma longa*	Korea	cellular renewal, regeneration, and antiaging

## Data Availability

Data are contained within the article.
